# Variable salinity responses of 12 alfalfa genotypes and comparative expression analyses of salt-response genes

**DOI:** 10.1038/srep42958

**Published:** 2017-02-22

**Authors:** Devinder Sandhu, Monica V. Cornacchione, Jorge F. S. Ferreira, Donald L. Suarez

**Affiliations:** 1 USDA-ARS US Salinity Lab, 450 W Big Springs Road, Riverside, CA 92507, USA; 2 INTA-Estación Experimental Agropecuaria Santiago del Estero, Jujuy 850, Santiago del Estero 4200, Argentina

## Abstract

Twelve alfalfa genotypes that were selected for biomass under salinity, differences in Na and Cl concentrations in shoots and K/Na ratio were evaluated in this long-term salinity experiment. The selected plants were cloned to reduce genetic variability within each genotype. Salt tolerance (ST) index of the genotypes ranged from 0.39 to 1. The most salt-tolerant genotypes SISA14-1 (G03) and AZ-90ST (G10), the top performers for biomass, exhibited the least effect on shoot number and height. SISA14-1 (G03) accumulated low Na and Cl under salinity. Most genotypes exhibited a net reduction in shoot Ca, Mg, P, Fe, and Cu, while Mn and Zn increased under salinity. Salinity reduced foliar area and stomatal conductance; while net photosynthetic rate and transpiration were not affected. Interestingly, salinity increased chlorophyll and antioxidant capacity in most genotypes; however neither parameter correlated well to ST index. Salt-tolerant genotypes showed upregulation of the *SOS1, SOS2, SOS3, HKT1, AKT1, NHX1, P5CS1, HSP90.7, HSP81.2, HSP71.1, HSPC025, OTS1, SGF29* and *SAL1* genes. Gene expression analyses allowed us to classify genotypes based on their ability to regulate different components of the salt tolerance mechanism. Pyramiding different components of the salt tolerance mechanism may lead to superior salt-tolerant alfalfa genotypes.

Water is the most limiting factor in modern agriculture. The current trend in increasing salinization of water resources and agricultural lands, the reduced availability of irrigation water of low salinity, and intense competition among different sectors (such as agriculture, urban and industrial) for water, makes the use of alternative or degraded waters inevitable. Different water sources that can be utilized include treated urban effluents and saline waters^1^. The most important consideration for the use of alternative/degraded water is often its salt concentration.

Salinity is one of the most important abiotic stresses that adversely affect plant growth and productivity globally^2^. About 1/5^th^ of the irrigated land used for agriculture is affected by salt^3^. In addition, the use of poor quality water for irrigation intensifies the salinity problem. Salt tolerance is the ability of a plant to tolerate higher concentrations of salt that can be measured in terms of maintenance of vigor, sustained growth, and yield. Salinity affects plants in two important ways: through osmotic stress and/or ionic stress^2^,^4^. To cope with salinity stress plants develop physiological and biochemical responses, and ecological strategies for either avoiding or tolerating the stress. Some common strategies include, ion uptake by the roots, ion exclusion from the roots, ion accumulation in vacuoles of root or shoot cells, regulation of ion transport from root to shoot, increased tolerance to high concentrations of toxic ions, and accumulation of compatible solutes^2^,^5^. In order to tackle this complex problem of plant salt tolerance, it is important to link the biochemical and physiological responses with the underlying genetic mechanisms. The identification of genetic mechanisms regulating salt tolerance is the key in developing genetic material tolerant to salt.

In the last decade some progress has been made in understanding physiological reactions involved in salt tolerance. However, characterization of molecular and biochemical responses in crop plants is still in its infancy as most of these responses are determined in model plant systems^6^. Molecular mechanisms regulating salt tolerance in moderately salt tolerant species, such as alfalfa, are expected to differ in some aspects. Hence, characterization of alfalfa genes regulating mechanisms involved in salt tolerance is crucial for both selection and breeding of salt-tolerant cultivars.

A large number of proteins involved in ion exclusion, ion compartmentalization, detoxification of effects of accumulated ions and regulation of gene expression are induced in plants exposed to salinity. Some alfalfa genes have provided increased tolerance in *Arabidopsis* against salinity. For example, transformation of alfalfa ethylene response factor gene (*MsERF11*) led to enhanced tolerance to salt in Arabidopsi*s*^7^. Overexpression of the transcription factor *Alfin1* boosted expression of the *MsPRP2* gene and increased salt tolerance in alfalfa^8^. Genes involved in the synthesis of polyols, sugars, proline and betaines that control homeostasis and play important role in maintaining osmoregulation during salt stress, have been explored in plants^9^,^10^. The antioxidant glutathione and amino acid proline have been recently cited to act concertedly to allow plants to withstand the joint attack of metalloids and salinity^11^.

Reactive oxygen species (ROS) are produced in plants as byproducts of normal metabolic reactions such as photosynthesis and respiration^12^. However, ROS are overproduced in response to abiotic (salinity, drought, etc.) or biotic stresses, leading to a dangerous imbalance between oxidants/antioxidants^13^. Antioxidants are then produced inside cells to remove ROS and to allow the plant to balance its oxidant/antioxidant status. These detoxifying antioxidants can be enzymatic (superoxide dismutase, catalase, peroxidase, etc.) or non-enzymatic (glutathione, ascorbic acid, vitamin E, phenolics, etc.)^14^. Expression analyses have been performed for many plant genes involved in synthesis of detoxifying enzymes and used to understand the importance of these genes in salt tolerance^5^. Additionally, it is appropriate to use analytical tests that can detect and quantify the general differences in antioxidant capacity among alfalfa genotypes before searching for the specific compounds responsible for the change in antioxidant capacity triggered by salinity stress. Some of these tests are the oxygen radical absorbance capacity (ORAC) and the total phenolics (TP) test using the Folin-Ciocalteu reagent^15^,^16^.

Traditional breeding programs for salt tolerance had limited success, in part because selecting plants based only on shoot biomass in saline conditions results in selection for plant vigor. Selecting individual plants for both vigor and purported attributes of Na and Cl ion exclusion in the presence of salinity may improve the potential to obtain clonal plants that are high yielding and more salt tolerant than the parents. Furthermore, a variety of forward and reverse genetic tools have been developed for the functional characterization of alfalfa genes in the last decade. *Medicago truncatula* genome has been sequenced and an RNAseq atlas has been developed for alfalfa^17^,^18^. Although putative genes have been predicted based on the DNA sequence, and annotated for possible functions based on protein homology, the functional characterization of genes involved in salt tolerance is still lacking. Recent studies show that there is considerable variation for salt tolerance in alfalfa genotypes^19^,^20^. However, the link between variation for salt tolerance and the genetic mechanisms leading to that variation is essentially missing. A combination of physiological, biochemical, and genetic approaches may help in selecting salt tolerant genotypes that are vigorous and high yielding.

## Results

In a previous study, 160 plants were selected from four different treatments consisting of two salinity levels of irrigation water (EC_iw_ 18.3 and 24.5 dS m^−1^) and two combinations of dominant salt types (Cl or SO_4_) in the irrigation water^20^. Of these 160 plants, 12 were selected based on biomass yield and ion (Na^+^ and Cl^−^) accumulation, and were classified as high, medium and low (Table 1). These 12 plants/genotypes were assigned Genotype numbers (G01 through G12) for convenience {SISA 15 (G01), Cuf 101-1 (G02), SISA 14-1 (G03), Cuf 101-2 (G04), SW9720 (G05), SISA 9 (G06), SISA 14-2 (G07), SISA 14-3 (G08), SISA 10 (G09), AZ-90 ST (G10), SW9215 (G11), Salado (G12)}. Subsequently, selected plants were vegetatively propagated as clones for subsequent experiments. These clones maintained the genetic characteristics of their parent plants in regards to being high, medium, or low in biomass and ion accumulation. Under the controlled greenhouse growth environment, this genetic similarity allowed us to focus on the effects of salinity on biomass accumulation and genetic expression, and eliminated unwanted plant-to-plant variation.

### Effect of salinity on shoot biomass of the alfalfa genotypes

**Figure 1:**
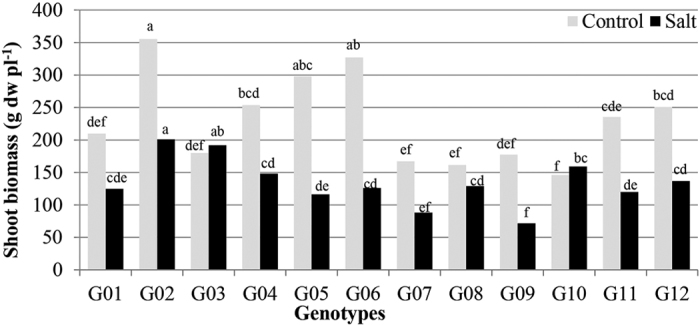
Total shoot biomass per plant of 12 alfalfa genotypes. Genotypic means for a treatment followed by the same letters are not significantly different according to LSD (0.05). SISA 15 (G01), Cuf 101-1 (G02), SISA 14-1 (G03), Cuf 101-2 (G04), SW9720 (G05), SISA 9 (G06), SISA 14-2 (G07), SISA 14-3 (G08), SISA 10 (G09), AZ-90 ST (G10), SW9215 (G11), Salado (G12).

Highly significant effects were found for treatments, genotypes and their interactions on biomass (Table 2). Salinity impact on plant growth was genotype dependent. Plant growth was reduced in most genotypes under saline conditions, with a maximum reduction of 61% in G06 (Fig. 1). G03 and G10, however, did not exhibit any reduction in biomass (Fig. 1). Genotypes G02, G03 and G10 ranked first, second and third, respectively, in shoot biomass under salinity stress (Fig. 1). G09 produced the lowest amount of biomass under salinity stress.

**Figure 2:**
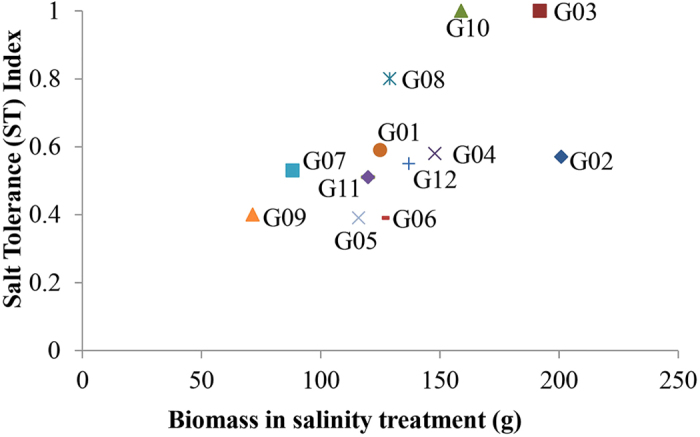
Relationship between total biomass in salinity treatment and salt tolerance of the alfalfa genotypes. Each point represents the mean value of six replicates.

Salt tolerance (ST) index, the ratio of performance under salt stress to performance under controlled condition, for the studied genotypes ranged from 0.39 (G05 and G06) to 1.0 (G03 and G10) (Fig. 2). The biomass production under salt stress displayed a significant positive correlation (*P* < 0.001, r = 0.67) with salt tolerance. The most salt tolerant clones G03, followed by G10 and G08 were among the top yielders under saline conditions (Fig. 2). The performances of these genotypes were consistent with their mother plants as all three mother plants had high biomass and low Na and Cl shoot concentrations (Table 1).

### Effect of salinity on height and shoot number

**Figure 3:**
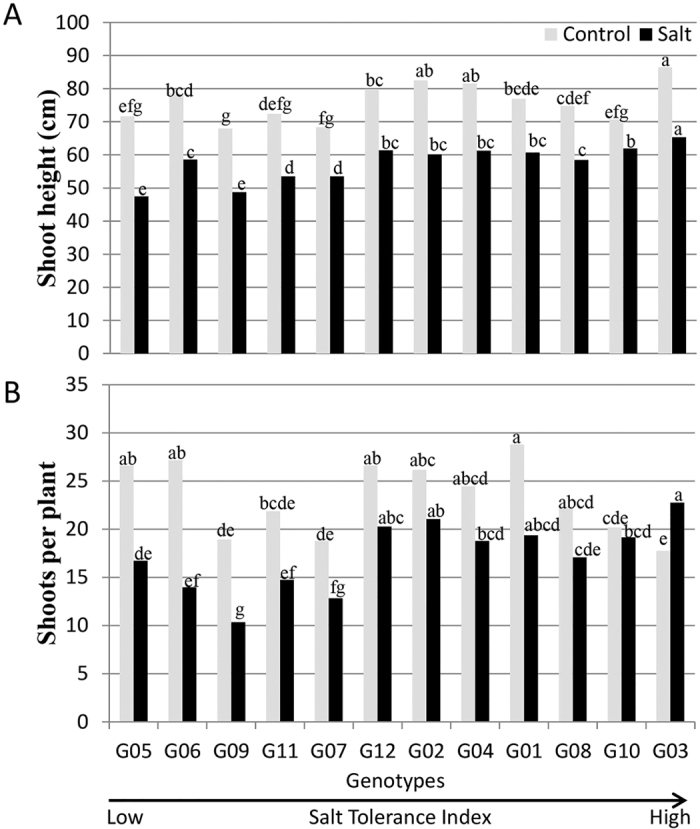
Shoot height (**A**) and shoots per plant (**B**) of 12 alfalfa genotypes. Genotypic means (n = 6) for a treatment followed by the same letters are not significantly different according to LSD (0.05). Genotypes are arranged on *x*-axis in ascending order based on their ST index.

Highly significant effects were found for treatments, genotypes and their interactions on height and shoot number per plant (Table 2). Accumulated biomass exhibited positive correlations with height (r = 0.71, n = 144) and shoot number (r = 0.87, n = 144). Salinity decreased shoot height in different genotypes from 12% to 34%, compared to the control (Fig. 3). The genotype with the lowest ST index (G05) was also the shortest with maximum reduction in plant height (Fig. 3). Similarly, G09 was the lowest biomass accumulator, low in ST index, and one of the shortest with the fewest shoots under salinity (Fig. 3). G03 that had no decrease in biomass, was the tallest, and exhibited an increased number of shoots under salinity (Fig. 3). G10 had the least salinity effect on height and displayed minimal decrease (~5%) in number of shoots (Fig. 3). Genotypes G03, G10, G02, G04, G08, and G12 showed less than 25% reduction in plant height and number of shoots under salinity (Fig. 3).

### Effect of salinity on shoot ion composition

**Figure 4:**
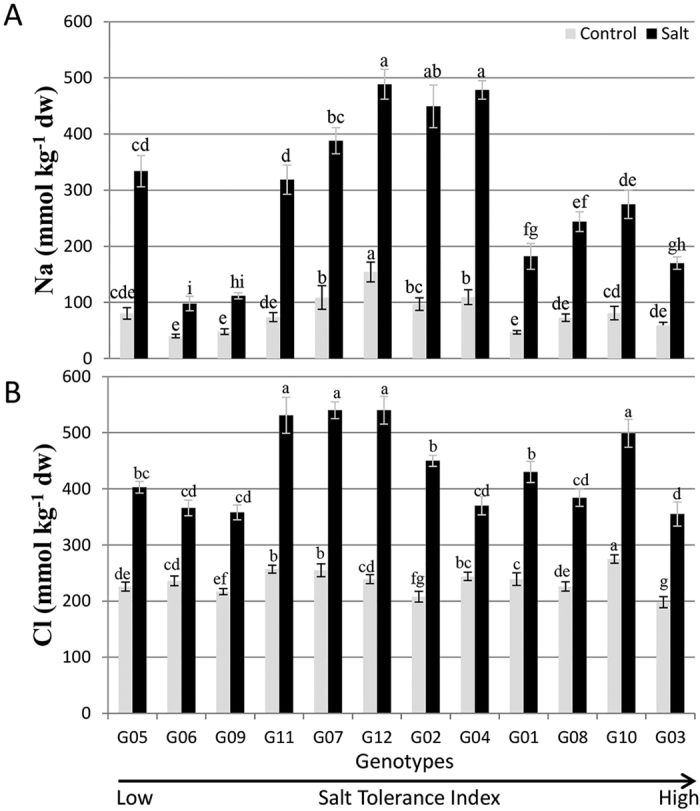
Shoot Na (**A**) and Shoot Cl (**B**) concentrations of 12 alfalfa genotypes. Genotypic means ± S.E. (n = 6) for a treatment followed by the same letters are not significantly different according to LSD (0.05). Genotypes are arranged on *x*-axis in ascending order based on their ST index.

The treatments, genotypes and the interaction effects were highly significant for the concentrations of all shoot ions (Table 2). All genotypes exhibited increased Na and Cl concentrations in shoots under salinity, but varied in the extent of that increase (Fig. 4). The average shoot Na concentrations ranged from 40 to 155 mmol kg^−1^ dw for plants under control and from 98 to 488 mmol kg^−1^ dw for plants under salinity treatments. Compared to the control, different genotypes under salinity treatments increased their Na concentration from 2.5-fold to 4.6-fold. G09 and G06 exhibited minimum increase in Na under salt treatment. G03, which showed high ST index, displayed only a 2.9-fold Na increase under salinity treatment. Although G12 had the highest shoot Na concentration under salt treatment, it was one of the genotypes with a relatively small Na increase (3.2-fold) under salt stress (Fig. 4). Among all the genotypes, G02 had the largest increase (4.6-fold) in shoot Na concentration under salt treatment, followed by G04 (4.4-fold) and G11 (4.3-fold) (Fig. 4A).

Shoot Cl concentration ranged from 198 to 275 mmol kg^−1^ dw for control plants and from 355 to 540 mmol kg^−1^ dw for plants under salinity (Fig. 4B). G03 stored the least amount of Cl under both control and salt treatments. G04 had the least (1.5-fold) increase in Cl concentration under salt treatment, G12 had the largest (2.3-fold) increase (Fig. 4B).

**Figure 5:**
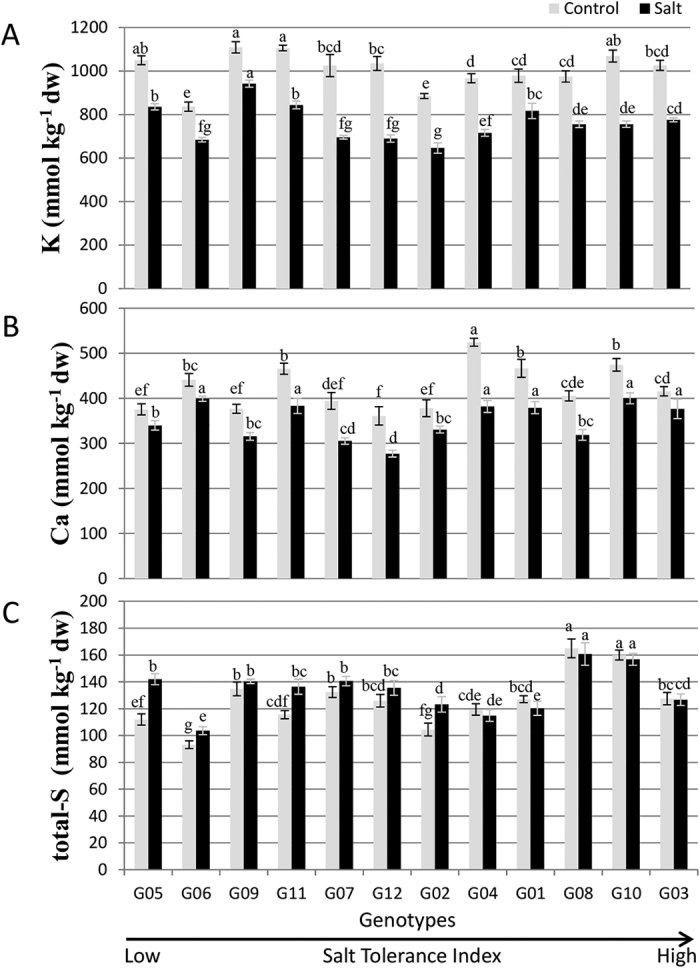
Shoot K (**A**), shoot Ca (**B**) and shoot S (**C**) concentrations of 12 alfalfa genotypes. Genotypes means ± S.E (n = 6) for a treatment followed by the same letters are not significantly different according to LSD (0.05). Genotypes are arranged on *x*-axis in ascending order based on their ST index.

On average, shoot K concentration decreased by 24% under salinity treatment compared to the control (Fig. 5A). G09 maintained the highest level of K and presented the least decrease in K (15.1%) under salinity. On the other hand, G12 had the maximum (33.4%) decrease in K content under salinity (Fig. 5A). The lowest K concentration under salinity was found in G02 followed by G06. Genotypes, G09 and G06 exhibited the highest K/Na ratio under salinity stress due to low Na concentration in the shoots (Supplementary Table S1).

Salinity significantly reduced (17.2%) shoot Ca concentration (Fig. 5B). Reduction ranged from 9.4% in G03 to 27.2% in G04. G10 had the highest Ca concentration (400 mmol kg^−1^) in the salt treatment.

There was little effect of salinity on total-S (6% reduction; Fig. 5C). The highest S concentrations were observed in G08 and G10, and the lowest in G06, under both treatments. Salinity also reduced the shoot P concentrations by 8% (Supplementary Table S1).

Overall, salinity reduced shoot Mg of genotypes by 7.5% (Supplementary Table S1). The Mg shoot concentration varied from 119 to 173 mmol kg^−1^ in the control and 99 to 180 mmol kg^−1^ in the salt treatment. Similarly, under salt stress, genotypes exhibited an average reduction of 14.3% and 5.7%, in shoot Fe, and Cu concentrations, respectively (Supplementary Table S1). On the other hand, under salt stress there were increases of 66.6% and 25.3% in shoot Mn and Zn concentrations, respectively (Supplementary Table S1).

The shoot ion concentrations (except for Mg and P) and biomass accumulation over all genotypes and treatment were significantly correlated (n = 144, *P* < 0.0001). The highest negative correlations between biomass and shoot ion concentration occurred with Cl (r = −0.499), S (r = −0.454), and Na (r = −0.389). ST index was also significantly correlated with shoot Cl (r = −0.401) and shoot Na (r = −0.388).

### Effect of salinity on gas exchange, foliar area and chlorophyll content

Net photosynthetic rates (*Pn*) and transpiration (*Tr*) did not show any significant change in the salt treatment as compared to the control in most genotypes; however, there was considerable variation among genotypes (Supplementary Fig. S1). Stomatal conductance (*gs*) showed consistent decrease in all genotypes in the salt treatment; although only few were statistically significant (Supplementary Fig. S1). G12 and G05 had the least reduction in the three parameters in the salt treatment as compared to the control. In contrast, the genotypes G02 and G03 performed the worst for these three parameters (Supplementary Fig. S1).

All genotypes exhibited a significant decrease in foliar area under salinity, except G03, G06, G10 and G11 (Supplementary Fig. S1). Salinity increased the chlorophyll content in most genotypes (Supplementary Fig. S1).

### Antioxidant capacity in response to salinity

Antioxidant capacity of all 12 genotypes increased as measured by the total phenolics (TP) and the ORAC tests (Supplementary Fig. S2). In general, TP ranged from 3.2 to 4.2 mg GAE g^−1^dw from control to salinity, while ORAC ranged from 100 (control) to 270 (salinity) μmoles TE g^−1^ dw. All the genotypes increased in ORAC levels under high-salinity irrigation water, with the maximum levels reported for G04 and the minimum levels for G10 (Supplementary Fig. S2A). Although, ten of the 12 genotypes had increased TP values, the differences were not significant between the control and high salinity irrigation water (Supplementary Fig. S2B). Only the hydrophilic antioxidant activity measured by the hydrophilic ORAC test resulted in significant increases in response to salinity.

### Expression Analyses

A set of 21 genes known to be involved in salt stress response in model plant species, was used for the expression analyses. The genes were selected based on functional conservation with the genes identified in model plants such as Arabidopsis and *Medicago truncatula*. The identified genes were classified into five groups based on different mechanisms of salt tolerance, as follows:

#### Genes involved in Na efflux from root to soil

**Figure 6:**
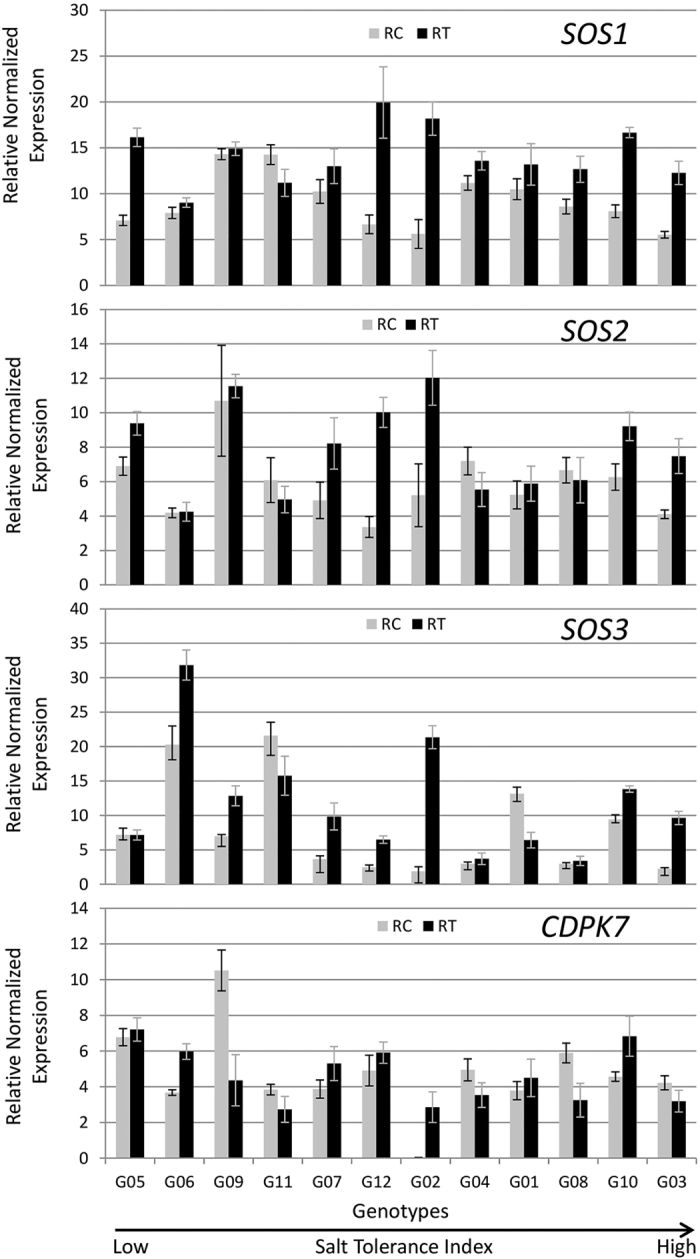
Expression of genes involved in Na efflux from root to soil in 12 alfalfa genotypes under saline and control treatments. Genotypes are arranged on *x*-axis in ascending order based on their ST index. RC, Control Roots; RT, Treatment Roots. Error bars represent standard error.

We studied the expression of four genes (*SOS1, SOS2, SOS3* and *CDPK7*) that are known to play important roles in Na efflux from root to soil. Genotypes G02 and G10 showed significant upregulation in roots for all four genes in salt treatment as compared to the control (Fig. 6). G03 and G12 displayed upregulation for *SOS1, SOS2* and *SOS3* but not for *CDPK7*. G06 presented upregulation only for *SOS1, SOS3* and *CDPK7* (Fig. 6). For *SOS1*, transcript accumulations in salt treatments were more than two-fold higher than control in G02, G03, G05, G10 and G12 (Fig. 6). For *SOS2*, G02 and G12 showed at least two-fold expression under salinity, compared to the control. For *SOS3*, G02, G03, G07, and G12 had over two-fold expression, while only G02 had over two-fold expression for *CDPK7* (Fig. 6).

Genotype G11 exhibited significant downregulation for all four genes involved in regulating Na efflux from root to soil in the roots of salt-treated plants (Fig. 6). Genotypes G03, G04, G08, G09 and G11 showed significantly reduced expression for *CDPK7* in roots of salt treated plants.

#### Sequestration of Na in vacuoles

Five important genes known to be involved in the sequestration of Na in vacuoles are *NHX1, NHX2, ATPase, SKIP1* and *AVP1*. These genes may play a role in partitioning excess Na in root and leaf vacuoles.

**Figure 7:**
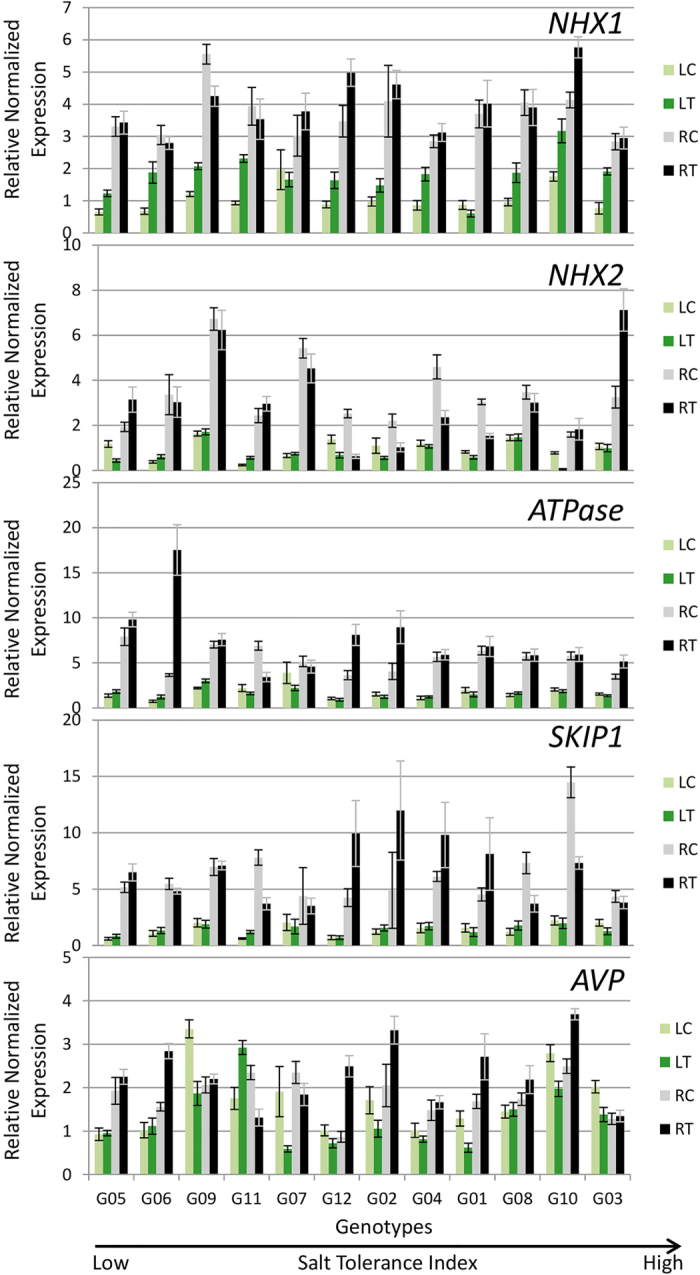
Expression of genes involved in sequestration of Na in vacuoles in 12 alfalfa genotypes under saline and control treatments. Genotypes are arranged on *x*-axis in ascending order based on their ST index. RC, Control Roots; RT, Treatment Roots; LC, Control Leaves; LT, Treatment Leaves. Error bars represent standard error.

Only one gene out of five involved in sequestration of Na in vacuoles was upregulated in salt treated roots of G03 (Fig. 7). All five genes were strongly induced by salt in roots of G12 plants. The *NHX1* gene was significantly upregulated only in G10 and G12 roots during salt stress; however, in leaves, it was significantly upregulated in 9 of the 12 genotypes (Fig. 7). *ATPase* was over 4-fold and 1.7-fold upregulated in salt treated roots and leaves of G06, respectively (Fig. 7). In the salt treatment, G03 exhibited significant upregulation for *NHX1* in leaves and *ATPase* in roots. G10 showed significant upregulation for *NHX1* in leaves and *NHX1* and *AVP1* in roots. G01 and G07 showed significant downregulation in leaves for three genes out of five. The *AVP1* gene was downregulated in salt treatment in the leaves of G12. Interestingly, expressions of *NHX2* and *AVP1* were significantly higher in the salt treatment as compared to the control in the roots of G01.

#### Retrieval of Na from xylem

**Figure 8:**
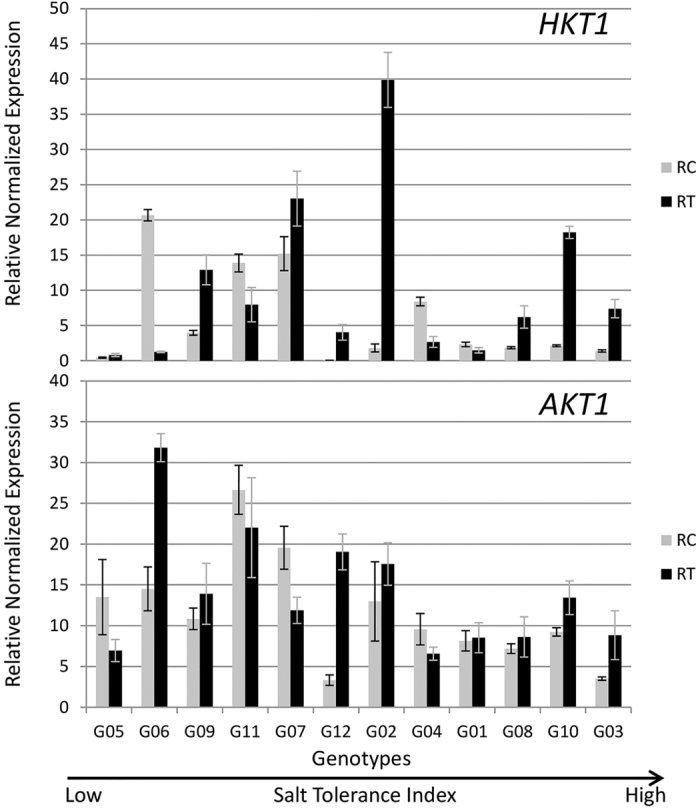
Expression of genes involved in retrieval of ions from xylem into the root in 12 alfalfa genotypes under saline and control treatments. Genotypes are arranged on *x*-axis in ascending order based on their ST index. RC, Control Roots; RT, Treatment Roots. Error bars represent standard error.

*HKT1* and *AKT1* are two genes known to play important roles in retrieving Na from xylem into root, thereby preventing salt damage to leaves. In G02, G03, G10 and G12, *HKT1* was expressed more than 5-fold in treatment roots as compared to the control (Fig. 8). *AKT1* was highly induced in treatment roots in G03, G06, G10 and G12. The *HKT1* gene was downregulated in G01, G04, G06, and G11 and the *AKT1* gene was downregulated in G04, G05 and G07 (Fig. 8). Both the genes were significantly downregulated in salt treated roots of G04.

#### Antioxidants and organic solutes

**Figure 9:**
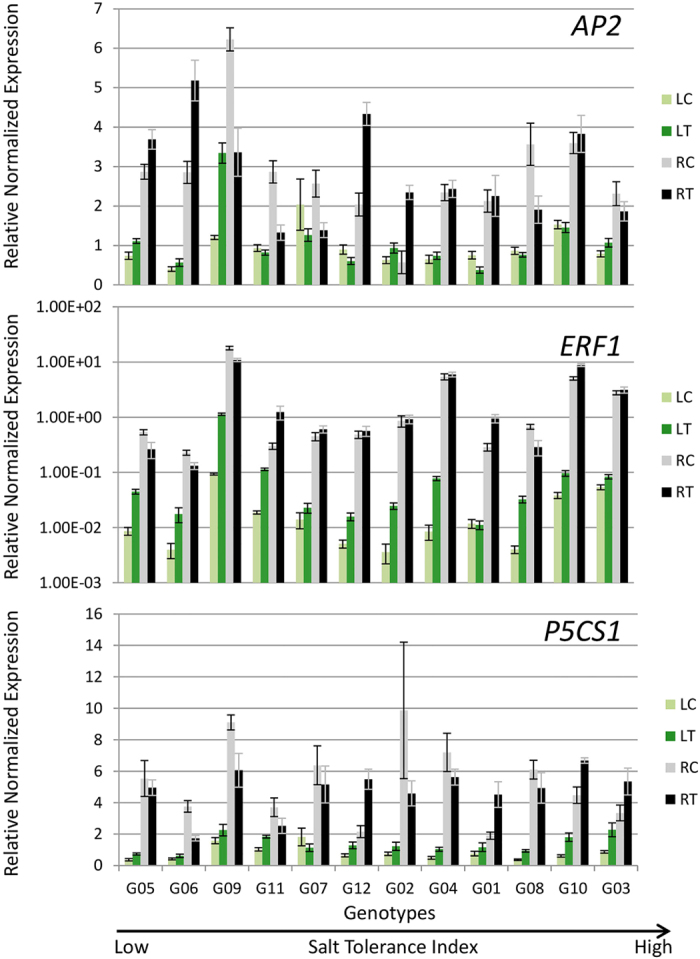
Expression of genes implicated in synthesis of antioxidants and organic solutes in 12 alfalfa genotypes under saline and control treatments. Genotypes are arranged on *x*-axis in ascending order based on their ST index. Due to large differences in the expression in leaves and roots in ERF1 bar diagram, Y-axis is presented in log scale. RC, Control Roots; RT, Treatment Roots; LC, Control Leaves; LT, Treatment Leaves. Error bars represent standard error.

In this study we also analyzed expression of the *AP2, ERF1* and *P5CS1* genes. *P5CS1* was significantly upregulated in salt treated roots of G01, G03, G10 and G12, while *ERF1* was upregulated in G01, G10 and G11 and *AP2* was upregulated in G02, G05, G06 and G12 (Fig. 9). In leaves, *ERF1* and *P5CS1* were significantly upregulated in all the genotypes except for G01 and G07. All three genes were significantly downregulated in G09. Interestingly, *AP2* was downregulated in G12 leaves of salt treated plants (Fig. 9). The *P5CS1* gene was over 2.5-fold upregulated in leaves of salt-treated G03, G08 and G10 plants.

#### Signal transduction

**Figure 10:**
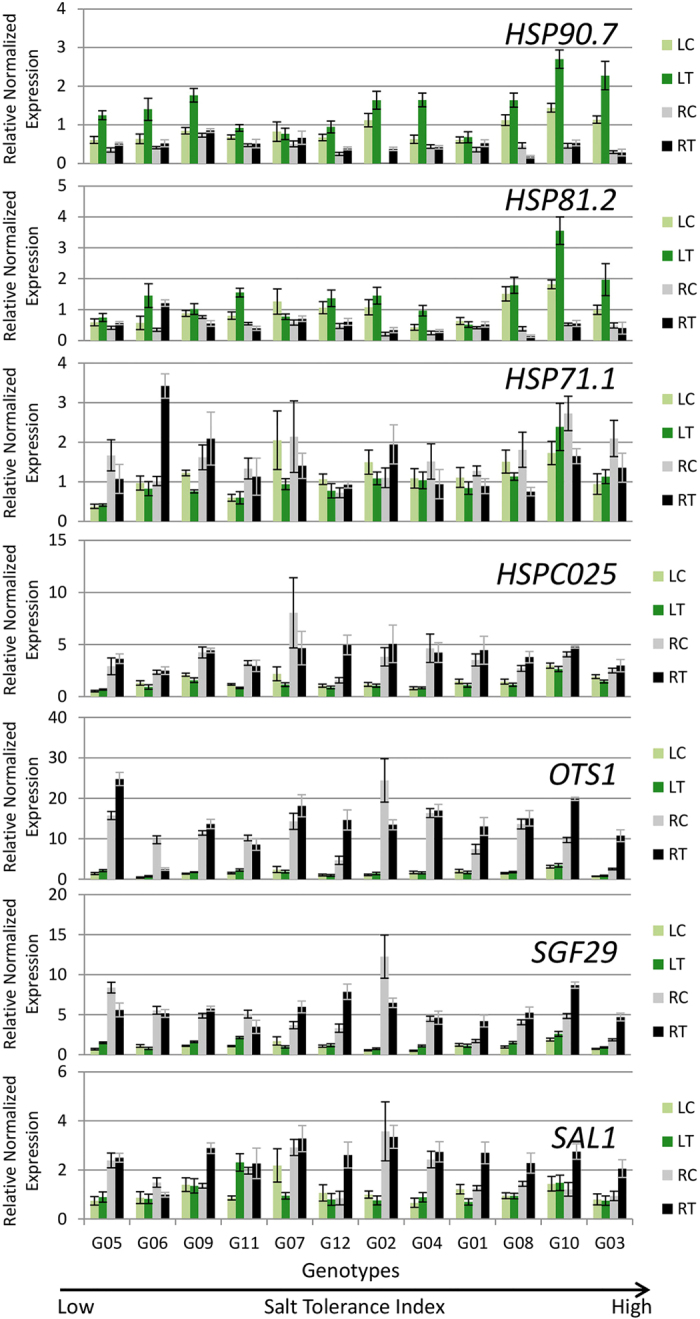
Expression of genes involved in salt related signal transduction events in 12 alfalfa genotypes under saline and control treatments. Genotypes are arranged on *x*-axis in ascending order based on their ST index. RC, Control Roots; RT, Treatment Roots; LC, Control Leaves; LT, Treatment Leaves. Error bars represent standard error.

Some genes that are known to be involved in signal transduction during salt stress were also investigated. These genes include heat shock protein genes (*HSP90.7, HSP81.2, HSP71.1* and *HSPC025*), overly tolerant to salt (*OTS1*), transcriptional activator SaGa Associated Factor 29 (*SGF29*), and *SAL1*. Heat shock protein genes, *HSP90.7, HSP81.2, HSP71.1* did not show significant upregulation in G03, G08, G10 or G12 in treatment roots (Fig. 10). Transcript levels of these three genes were significant reduced in G08 in treatment roots (Fig. 10). *HSP90.7* was upregulated in the salt treatment in leaves of several genotypes. However, in roots, G02 stands out with more than 1,200 fold upregulation in salt treatment. *HSPC025* showed significantly upregulation in salt treated roots of G08, G10 and G12 (Fig. 10). The genotypes G01, G03, G09, G10 and G12 displayed significant upregulation for all three genes, *OTS1, SGF29* and *SAL1* in salt treatment roots. *HSPC025, SGF29* and *SAL1* were significantly downregulated in G07 treatment leaves (Fig. 10).

The comparison of relative transcript levels in leaf and root tissues revealed high abundance in root as compared to leaf in all genes except *HSP90.7*and *HSP81.2* (Figs 6 to 10). Under salt stress conditions, the most abundant transcripts were those of the *HKT1* gene in root of G02 and the *HSP81.2* gene in leaf of G10.

## Discussion

This study was undertaken to understand the physiological and genetic basis of salt tolerance in alfalfa. Almost all salt tolerance screenings consider only biomass production under saline conditions. However, in our investigation we used 12 alfalfa genotypes previously selected for different levels of salt tolerance^20^, differences in Na and Cl concentrations and K/Na ratio in the shoots. As alfalfa is polyploid and highly heterozygous due to cross pollination, populations vary widely in their salt tolerance; in addition, there is a tremendous variability within populations^20^. To reduce variability within populations, we evaluated cloned individuals in this long-term salinity experiment.

Most of the genotypes exhibited reduction in biomass yield under salt stress (EC_iw_ = 16.6 dSm^−1^) with the exception of G03 and G10 (Fig. 1). Under salt treatment, shoot biomass yield displayed strong positive correlations with salt tolerance index, height and shoot number (Fig. 3), as reported previously^21^,^22^. High-performance consistency for the biomass yield between clonal plants with their parents confirmed the nature of the variation to be genetic (Table 1).

Salinity is known to reduce the concentrations of macronutrients (N, K, Ca and Mg) and micronutrients (Zn, Mn and Fe) in leaf tissue^23^. Ion accumulation in plant tissues is commonly used to evaluate the ability of a plant to tolerate salt. K/Na and Ca/Na ratios are important parameters that can be used for tolerance screening^24^. High salt concentrations lead to loss of K due to the depolarization of membranes and loss of Ca due to the displacement by Na ions^25^. Roots are known to be less affected by high salt concentrations due to transport of photosynthates to stressed roots to maintain osmotic balance^21^. On the other hand, leaves are more sensitive to salt and start showing toxicity much earlier than roots^26^. The genotypes that exclude salt from roots transport less salt to the aerial parts of the plants. In our investigation, leaf concentrations of Na, Cl, Mn and Zn increased, while K, Ca and Mg concentrations decreased under salinity (Figs 4 and 5 and Supplementary Table S1). Similar observations have been made previously for the forage legume *Lotus* spp.^27^,^28^ and *Medicago sativa*^10^. Salinity was also found to lead to the simultaneous increase in Na and decrease in K in shoots of *M. truncatula, M. laciniata*, and *M. polymorpha*; but the changes in Na and K were less accentuated in the latter two species that considered as more salt-tolerant than *M. truncatula*^29^. Although all 12 genotypes had decreased shoot concentrations of Ca under salinity, this decrease was not detrimental to plant development as the Ca concentration was high enough for shoot regrowth. Ca concentrations under salinity were also high enough to support the expression of the SOS gene pathway^30^, although a high SOS gene expression did not always correspond to a high concentration of shoot Ca (e.g., G02, Figs 5 and 6).

G03 stored least amount of Cl and displayed nominal increase in Na concentration in shoots (Fig. 4). Interestingly, G12, which presented less than 25% reduction for plant height and number of shoots, was the highest in Na and Cl concentrations under salt treatment (Fig. 4). Despite higher Na and Cl concentration in shoots, G12 was one of the high performers under salt treatment; suggesting that it is able to tolerate high concentrations of Na (Fig. 1). Our results for G12 contrast with previous observations that salt-tolerant glycophytes accumulate less salt than sensitive ones^28^,^31^. G02 displayed a 4.6-fold increase in Na concentration under salinity which may be one of the reasons for its low ST index (Figs 2 and 4).

High K/Na ratio has also been used as an indicator of salt tolerance in plants. A salt stress study on four alfalfa cultivars revealed that the cultivar with the highest K/Na ratio was the most tolerant to salt^24^,^32^. In our study, we did not observe any significant association between K/Na ratio and salt tolerance (Supplementary Table S1). The genotypes, G09 and G06 showed the highest K/Na ratios under both control and salt treatments, however, these genotypes were placed at the bottom of the ranking for ST index (Supplementary Table S1 and Fig. 2). On the other hand, G12, that was one of the high performers for shoot biomass, and had the lowest K/Na ratio in both treatments. These contrasting observations indicate that shoot Na and K concentrations represent only one aspect of the complex salt tolerance mechanism in alfalfa and cannot be used alone to establish if a plant is tolerant or sensitive to salt. In addition, selection based on a single salt salinizing solution such as NaCl, may lead to tolerance to specific inhibitory affects associated with that particular salt^33^. Several salt stress studies in plants were conducted using germination as a criterion for tolerance. However, salt tolerance of alfalfa genotypes at germination and post-germination do not show a strong correlation, suggesting that differences in tolerance mechanisms in germinating seeds and mature plants^21^,^33^. These findings highlight the importance of long-term salt stress studies spanning from young to mature plants and considering factors other than just Na and Cl tissue accumulation and K/Na ratio.

Gas exchange parameters are often used to determine overall performance of a plant. Transpiration rates can be affected by decreased K concentration in the leaf cells leading to stomatal closure^34^. Our observations are perfectly in alignment as G09 that maintained highest levels of K with minimum reduction in the salt treatment displayed both the highest values for *Tr* and *gs* under salt treatment (Fig. 5A and Supplementary Fig. S1). Interestingly, G02 and G03 were the bottom performers with respect to *Pn, Tr* and *gs*, collectively. These observations imply that although plants exhibited reduction in biomass accumulation and overall performance under salinity, gas exchange parameters may not directly contribute toward the poor performance under salinity. For *Pn*, there was no reduction with increased salinity (Supplementary Fig. S1A). This was expected, as *Pn* is often unchanged when the rates are expressed per unit leaf area, although *gs* is reduced^2^. Most genotypes displayed a decrease in leaf area under salinity (Supplementary Fig S1). Any reduction in the leaf area leads to a reduced *Pn* per plant^2^,^4^. In line with their performance in the salt treatment, two most salt tolerant genotypes G03 and G10 did not show significant decrease in leaf area. Also, salinity actually increased the chlorophyll content in all genotypes, contrary to what was recently reported after seven days of salt stress in alfalfa^35^.

Most genotypes had increased ORAC and TP values under salinity treatment as compared to the control, however, antioxidant activity did not correlate well with ST index. The main reason may be the complexity of the antioxidant response by the plant. Perhaps, there are multiple gene loci that regulate tissue antioxidant concentration under a particular environmental condition. Identification of specific antioxidants linked to salt tolerance may improve the knowledge of how antioxidant capacity relates to salt tolerance. Another reason may be that the genotypes studied in this investigation were preselected for salt tolerance and may not show a drastic increase as reported for salt sensitive genotypes.

In this study, we wanted to establish a relationship between genes involved in salt stress and phenotypic differences among different genotypes in response to salinity treatment. Thus, we evaluated the expression of candidate genes known to be involved in different mechanisms of salt tolerance including Na efflux from root to soil, sequestration of Na in vacuoles, retrieval of Na from xylem, antioxidants and organic solute synthesis and signal transduction.

**Figure 11:**
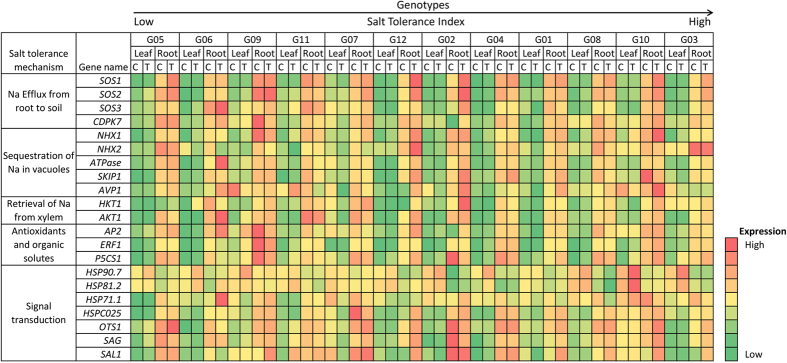
Change in expressions of salt-related genes in roots and leaves of alfalfa genotypes subjected to salt stress. Relative abundance of putative salt-stress genes in the control or salt stress treatments in roots and shoots of alfalfa genotypes. Expression values are color coded to depict the fold change. Genotypes are arranged on *x*-axis in ascending order based on their ST index.

Plant salt tolerance is regulated through several different pathways including salt overly sensitive (SOS) pathway^36^. The *SOS1, SOS2* and *SOS3* genes are directly involved in regulating salt tolerance by excluding Na from plant roots. SOS3 is activated by Ca binding and interacts with SOS2 to activate the kinase, which in turn phosphorylates SOS1^5^. Phosphorylation increases transport activity of SOS1, a plasma membrane associated protein that controls Na efflux from the root^37^. CDPK7, a calcium-dependent protein kinase that regulates Na efflux, is also shown to provide salt tolerance in rice^38^. The SOS genes were upregulated in tolerant genotypes suggesting that Na exclusion is an important factor in providing salt tolerance in these genotypes (Fig. 11). Strong Na exclusion mechanism has been shown to be the key for the enhanced salt tolerance in several plant species^2^,^32^. Overexpression of SOS proteins has been shown to provide enhanced tolerance to salt in heterologous species^32^,^39^,^40^.

Several genes have been identified in different plant species that are known to play important roles in sequestering Na into the vacuoles to protect cytosol from the toxic effects. NHX1 and NXH2 are two isoforms that are present in the vacuolar membrane and partition Na into the vacuole^41^. *NHX1* was significant upregulated in the salt treatment in many genotypes in leaves, whereas, in roots it was upregulated only in G10 and G12 (Fig. 11). These observations suggest that NHX1 may play a primary role in partitioning excessive Na in leaf vacuoles; however, additional partitioning in root vacuoles may present an added advantage for some genotypes.

HKT1 is involved in retrieving Na from xylem into root that keeps the sodium levels low in the leaves^42^. The *HKT1* gene was upregulated more than 64-fold in G12 and more than 22-fold in G02 roots. High expression of the *HKT1* gene in G02 may be one of the reasons for its highest biomass yield under salinity (Fig. 1).

Antioxidants and organic solutes play important roles in increasing tolerance of plant tissue to excessive salt concentrations. It has been known that plants accumulate antioxidants in response to salinity to neutralize elevated levels of ROS^43^. Plants that are able to increase their tissue antioxidants may have a higher tolerance to salinity^14^. *AP2* encodes ascorbate peroxidase, which has a role in scavenging ROS^44^. *ERF1* is activated by salt-induced ROS^43^. *P5CS1* codes for pyrroline-5-carboxylate synthetase that is involved in proline synthesis and accumulation during salt stress^45^. Considerable variation has been detected in crop plants for different antioxidants and osmoprotectants^13^,^14^,^46^,^47^.

*P5CS1* was 2.5-, 2.4-, 1.6- and 1.5-fold upregulated under saline conditions, as compared to the control, in G12, G01, G03 and G10 roots, respectively (Fig. 9). On the other hand, salt sensitive genotypes G09, G11 and G06 had a drastic decrease in *P5CS1* expression in salt treatment. In leaves, genotypes G03, G08 and G10 displayed at least a 2.5-fold upregulation for *P5CS1*, although most other genotypes also showed upregulation. Higher expressions of *P5CS1* in roots and leaves of salt tolerant genotypes in saline conditions suggest the importance of proline accumulation in salt tolerance. In addition to synthesis of proline by *P5CS1*, silencing of proline dehydrogenase, an enzyme involved in degradation of proline, is also known to provide tolerance to salinity through the accumulation of proline^9^.

The ortholog of ERF1 were shown to provide better protection from damage to cell membranes and ultimately, better tolerance to salinity^7^,^48^. However, in our investigation salt tolerance did not show any significant association with *ERF1* expression in roots. In leaves, *ERF1* was upregulated in most of the genotypes. The *CaZF* gene that plays an important role in salinity tolerance was regulated by *AP2*^49^. *AP2* showed upregulation in roots of G02 and G12, two high performing genotypes in salt with similar salt tolerance (Figs 2 and 11).

Heat shock proteins are known to play important roles in various environmental stresses including salinity stress^50^. *HSP90.7, HSP81.2*, and *HSP71.1* did not show upregulation in roots of tolerant genotypes suggesting that their expression in alfalfa roots may not be critical for salt tolerance (Fig. 11). However, *HSPC025, OTS1, SGF29* and *SAL1* were upregulated in roots of salt tolerant genotypes (G03, G10, G08 and G12) (Fig. 11). G02 did not show upregulation for *OTS1, SGF29* and *SAL1* in roots, which may be one of the reasons for a sharp decline in performance of this genotype under salinity. Expression patterns of *OTS1, SGF29* and *SAL1* were very similar in all salt tolerant genotypes, suggesting their involvement in same physiological process (Fig. 11).

Most of the genes exhibited significant induction in roots or leaves under salinity stress; however upregulation in response to salt treatment was more commonly seen in roots than in leaves. Similar observations were made in Arabidopsis where several regulatory genes indicated upregulation in roots exposed to salt treatment whereas shoots showed upregulation under osmotic stress^51^. Also, *M. truncatula* exposed to salt stress for 12 and 24 hours presented higher expression of the *flavanone 3 hydroxylase* gene in roots compared to leaves and stems^52^. Seven genes *SOS1, SOS2, SOS3, HKT1, OTS1, SGF29*, and *SAL1* were consistently upregulated in the roots of the genotypes that performed well for biomass yield and physiological parameters (Fig. 11). Similarly, in leaves, five genes that were consistently upregulated in better performing genotypes were *NHX1, ERF1, P5CS1, HSP90.7*, and *HSP81.2* (Fig. 11). Although functional relevance of many of these genes is not completely understood, several transcripts displayed predominant specificity to salinity stress, perhaps due to their role in plant acclimation to stress.

Genotypes G03 and G10 that displayed the highest salt tolerance, exhibited upregulation of the *SOS1, SOS2, SOS3, HKT1* and *AKT1* genes suggesting that G03 and G10 are highly efficient in Na efflux from the root and retrieval of Na from xylem (Fig. 11). These observations are justified by Na accumulation data as G03 and G10 displayed only 2.9-fold and 3.4-fold increases in Na concentration in salt treatment, respectively and are among low Na accumulators (Fig. 4). Both genotypes showed upregulation for *NHX1* in leaves that is involved in sequestration of Na in vacuoles and *P5CS1* that is involved in accumulation of proline. Hence, these genotypes may exhibit better tissue tolerance. Furthermore, G03 and G10 also show upregulations for some signal transduction genes such as *HSP90.7, HSP81.2, HSP71.1* in leaves and *HSPC025, OTS1, SGF29* and *SAL1* in roots. Gene expression data are in agreement with high ST index, high shoot biomass accumulation and the ability of G03 and G10 to accumulate low Na concentrations in shoots.

All 4 genes involved in Na efflux and *HKT1* were highly upregulated in the salt treatment in G02, the genotype that produced the most biomass in the control and in salinity treatments (Figs 6, 8 and 11). These observations suggest that this genotype is expected to be good in excluding Na from the root and also in retrieving Na from the xylem. However, the analysis of data for Na concentration in shoots revealed that G02 presented the largest increase under salinity (Fig. 4). These findings indicate that it is likely that there are additional genes that may regulate either exclusion of Na from roots or transport of Na from roots to shoots. Members of other gene families such as *NHA* (Sodium-Proton Antiport), *CHX* (Cation H^+^ Exchanger) or *NSCs* (Non-Selective ion Channels) may also play an important role in regulating Na concentration in leaves^53^. On the other hand, due to lack of upregulation of *NHX1* and *NHX2*, G02 is not able to sequester Na into the vacuoles in roots. In addition, *P5CS1*, the gene involved in accumulation of proline is not upregulated, leading to low tissue tolerance of this genotype, which explains its low salt tolerance index of 0.57 (Fig. 2).

In this investigation we are able to differentiate genotypes based on different components of salt tolerance mechanism. Combining attributes from different genotypes by crossing and selecting for lines with multiple components of salt tolerance mechanism may help in developing alfalfa lines with enhanced salt tolerance. In addition, this knowledge may help in isolating genes or quantitative trail loci important in salt tolerance.

## Material and Methods

### Selection of the mother plants and cloning

In a previous experiment, we ranked alfalfa plants based on their biomass accumulation recorded in nine harvests from tanks irrigated with high-salinity water^20^. These waters had electric conductivity (EC_iw_) of 18.3 and 24.5 dS m^−1^ and were dominant in either Cl or SO_4_. We identified plants with high and low biomass accumulation from each of the four treatments, with a total of 160 plants^20^. Shoot samples (leaves + stems) were taken from each of 160 plants and analyzed separately for ion composition. The results were ranked for biomass accumulations and data were averaged for high biomass and low biomass producers for each treatment^20^. The ion content of each plant was also ranked as low, medium, and high accumulation of each ion.

Of total 160 plants, 12 were selected, and classified, based on their biomass accumulation (high, medium or low) (Table 1). Selected plants were also ranked as high, medium, or low shoot Na and Cl accumulators (Table 1). These 12 plants/genotypes derived from 9 different cultivars/breeding lines and were assigned Genotype numbers (G01 through G12) for convenience {SISA 15 (G01), Cuf 101-1 (G02), SISA 14-1 (G03), Cuf 101-2 (G04), SW9720 (G05), SISA 9 (G06), SISA 14-2 (G07), SISA 14-3 (G08), SISA 10 (G09), AZ-90 ST (G10), SW9215 (G11), Salado (G12)}. Two different plants/genotypes were selected from Cuf 101 and were named as Cuf 101-1 and Cuf 101-2, three different plants/genotypes were selected from SISA 14 and were named as SISA 14-1, SISA 14-2 and SISA 14-3 (Table 1). Of the 9 cultivars/breeding lines selected, Cuf 101, SW9720, SW9215, and Salado are non-dormant American cultivars, with SW9720, SW9215, and Salado considered as salt tolerant for forage production according to the latest report of Alfalfa Variety Ratings^54^. AZ-90 ST is a cultivar that is generally used as a standard for salt tolerance, according to the protocol for forage production under salinity stress. The other genotypes SISA 15, SISA 14, SISA 10, and SISA 9 were originally selected as breeding lines under different natural conditions of saline and saline-sodic soil in Santiago del Estero, Argentina, by INTA’s alfalfa breeding program through a cooperative project between Santiago del Estero and Manfredi Experiment Stations in Argentina. Selected plants were transplanted in different sand tanks and were irrigated with tap water supplemented with essential nutrients (EC_iw_ = 1.97 dS m^−1^). Plants were harvested every month for a few months and the biomass was discarded. Subsequently, selected genotypes were cloned via cuttings (about 10 cm long with two nodes) from each mother plant. Cuttings were dipped in Rootone^®^ to boost rooting. Clones were grown in the same tanks with their mother plants and under the same irrigation condition.

### Experimental setup for evaluation of 12 genotypes

The experiment was conducted in the greenhouse lysimeter system at United States Salinity Laboratory (USDA-ARS) in Riverside, CA {33°58′24″N latitude, 117°19′12″W longitude}. The experiment was set up as a completely randomized design with two treatments: control and saline water, with six replicates. Two plants per clone were planted together in each previously randomized sand tank (120 cm long by 60 cm wide by 50 cm deep). Twelve clones were planted in two rows (6 clones per row) in each tank.

Plants were irrigated once, daily, with a base nutrient solution made up with Riverside tap water (EC = 0.6 dS m^−1^): 3.4 mmol_c_ L^−1^ Ca^2+^, 0.8 mmol_c_ L^−1^ Mg^2+^, 1.6 mmol_c_ L^−1^ Na^+^, 0.1 mmol_c_ L^−1^ K^+^, 1.3 mmol_c_ L^−1^ SO_4_^2−^, 0.83 mmol_c_ L^−1^ Cl^−^, and 0.48 mmol_c_ L^−1^ NO_3_^−^. The base nutrient solution was a modified Hoagland’s solution consisting of the following micronutrients (in μmol L^−1^): Fe 50 μmol L^−1^ added as Fe-DTPA (Sprint 330^®^), ZnSO_4_.7H_2_O 0.4 μmol L^−1^, CuSO_4_.5H_2_O 0.2 μmol L^−1^, H_2_MoO_4_ 0.1 μmol L^−1^, H_3_BO_3_ 23 μmol L^−1^, and MnSO_4_ 5 μmol L^−1^; and macronutrients: KNO_3_ 5.0 mmol_c_ L^−1^, KH_2_PO_4_ 0.5 mmol_c_ L^−1^, CaCl_2_ 1 mmol_c_ L^−1^, and MgSO_4._7H_2_O 3.3 mmol_c_ L^−1^. The water pH ranged between 7.0 and 7.8. During the experimental period, additional KNO_3_ was added twice (~4.5 and 3 mmol_c_ L^−1^) to all reservoirs to maintain target concentrations of K^+^, NO_3_^−^, and EC_iw_. Irrigation solutions were prepared in 890 L reservoirs underneath the greenhouse and pumped through PVC pipes to irrigate sand tanks. The excess irrigation water returned to the reservoirs via drainage by gravity for reuse. The water lost by evapotranspiration was replenished in the reservoirs by adding deionized water to maintain a constant electrical conductivity of the irrigation water and in the sand tanks. The plants were sprayed with Enstar^®^, Safari^®^, and Floramite^®^, PyGanic^®^, and Strike^®^ to control thrips, mites, aphids, and powdery mildew as required.

### Salt treatment

Salt treatment was started 7 days after the rooted clones were transplanted. The salt treatment was imposed by adding specific amounts of MgSO_4_, Na_2_SO_4_, CaCl_2_, NaCl, and KCl, to tap water (plus nutrient solution) in increasing quantities every 7 days until the tank waters reached EC_iw_ = 16.6 dS m^−1^ after two weeks. The saline solution was dominated by Cl^−^ (approximately Cl^−^/SO_4_^2−^ = 2 when concentration is expressed in mmol_c_ L^−1^). EC_iw_ (for control and saline water treatment) was measured with an electrical conductivity meter once a week. The irrigation water compositions were averaged over samples taken during the experimental period (Table 3). Target salinity of irrigation waters was maintained until the final harvest.

### Biomass

The first cutting of the clones was discarded (March 2014) as plants were still adapting to incremental increase in salinity during the first month. Plant biomass was harvested 17 times monthly from April 2014 to October 2015, only the biomass of one harvest (June 2015) was not recorded. At harvest, all plants were cut approximately 5 cm above the crown. Plants fresh weight was recorded and, after drying at 70 °C for 48 h, dry weight (dw) was recorded for every harvest. Total shoot biomass per plant for each genotype was determined for 16 harvests. Height (cm) and number of shoots per plant were averaged across all harvests. The salt tolerance (ST) index was defined as the mean biomass of plants under salinity divided by the mean biomass of control plants. This ratio is referred to as relative biomass ratio, and it is a criterion usually used to measure salt tolerance^55^.

### Ion Composition

Shoot samples of each clone from each replication were taken at four different harvests (April 1, May 2, August 5 in 2014 and March 11, 2015). The samples were washed with deionized water immediately after harvesting, and dried in a forced-air oven at 70 °C for 72 h. Chloride was determined from nitric-acetic acid extracts by amperometric titration. The concentrations of Na, K, Mg, Ca, total-S and micronutrients (Fe, Cu, Mn, Zn, and Mo) were determined from nitric acid digestions of the dried, ground plant material by ICPOES. Each harvest was analyzed separately.

### Gas exchange measurements, leaf area and chlorophyll content

Gas exchange, leaf area, and chlorophyll were determined by taking measurements from each of two clones of each genotype in three replicates. The measurements were done in April 2014 just before the 1^st^ harvest utilizing fully-expanded 3^rd^ trifoliate from the top. Leaf net photosynthetic rate (*Pn*), leaf stomatal conductance (*gs*), and leaf transpiration (*Tr*) of middle leaflet of the trifoliate were measured using a portable Li-Cor 6400 Photosynthesis System. The measurement conditions were leaf chamber PAR (photosynthetically active radiation), 1100 μmol m^−2^ s^−1^; leaf to air vapor deficit pressure, 1.5 to 2.4 kPa; leaf temperature 23–28 °C and chamber CO_2_ 380 μmol mol^−1^. Because the leaflet is too small to cover the entire surface area of the 2 × 3 cm leaf chamber, its area needed to be estimated in order for the Li-Cor 6400 to calculate *Pn, gs* and *Tr*. An empirical formula for the area of a typical oval shape (0.8WL, W is the maximum width, L is the maximum length of the shape or leaflet) was adopted by correlating the values from 0.8WL with those from actual leaflet area measurement using a Li-Cor 3100 Area Meter. Thus, for each leaflet, both maximum width and length were measured and its area was estimated using the formula (alfalfa leaflet area = 0.0922 + 0.852 *0.8WL). Gas exchange was measured in the same leaflets using a portable Minolta Chlorophyll Meter, SPAD-502 (three readings from each leaflet). Calibrations of SPAD readings with leaf chlorophyll concentrations were performed previously. Thus, the concentrations were estimated using the formula {Chl (a + b) = −0.925 + 0.0950*SPAD value}.

### Oxygen Radical Absorbance Capacity (ORAC) and Total Phenolics (TP) Analyses

ORAC and TP were analyzed for 12 genotypes with 6 replications each. Ground dried samples (0.5 g) of alfalfa tops were mixed with 5 g of sand. Each mixture was then extracted in a pressurized stainless steel cell (ASE 350, Thermo Scientific/Dionex, Sunnyvale, CA, USA) using hexane to extract the lipophilic fraction and acetone:water:acetic acid (70:29.5:0.5 by volume) for the hydrophilic fraction. The extraction time was 5 min, followed by a 100% flush, a 60-s purge with 2 cycles, at 80 °C and 1500 psi. The hexane extract was evaporated to dryness with nitrogen in an evaporator (N-EVAP, Organomation, Berlin, MA, USA) at 37 °C and then redissolved in 10 mL of pure acetone; a 50-μL aliquot was collected for dilution and lipophilic ORAC analysis. After extraction with aqueous acetone by the ASE 350, the samples were made up to a volume of 25 mL in the acetone-water-acetic acid solution. A 150-μL aliquot of the aqueous acetone extracts was diluted for hydrophilic ORAC analysis. The ORAC assay is based on the inhibition of the peroxyl-radical-induced oxidation initiated by thermal decomposition of azo-compounds such as (2,2′-azobis (2-amidino-propane) dihydrochloride (AAPH))^15^. Samples were analyzed for their antioxidant capacity (ORAC) in triplicate. The same ASE 350 aqueous acetone extracts were used for quantification of TP according to the Folin-Ciocalteu method^16^,^56^ using gallic acid (cat. No. 398225, Sigma-Aldrich, Saint Louis, MO, USA) as the standard. A 20-μL aliquot of the extracts or a gallic acid standard solution was pipetted into a cell of a 96-cell microplate, followed by the addition of 100 μL of 0.4 N Folin Ciocalteu phenol reagent (stock solution F9252, Sigma-Aldrich, Saint Louis, MO, USA) and the addition of 80 μL of 0.94 M Na_2_CO_3_. The plate was covered with a plastic plate cover and allowed to develop color for 5 min at 50 °C. The absorbance was read at 765 nm using a microplate spectrophotometer (xMark™, BIO-RAD, Hercules, CA, USA).

### Statistical analyses

A two-way analysis of variance (ANOVA), with the treatment and genotype as factors, was performed for the whole data set using the InfoStat statistical program^57^. Spatial position inside the tanks (corner or between) was considered as a covariable when the parameters were significantly affected by the position. Means were compared using the Fisher’s Least Significant Differences (LSD) test at 5% level of significance. Partial correlations between two variables were adjusted for the spatial position when the position significantly affected the parameter. Differences in gas exchange measurements, leaf area, and chlorophyll content between the two treatments within a genotype were analyzed at p ≤ 0.05 using the T-test procedure.

### Primer design for expression analysis

Twenty-one genes involved in different mechanisms leading to salt tolerance were selected based on functional conservation with the genes identified in model plants such as *M. truncatula*, Arabidopsis and rice. Corresponding gene sequences were used in Basic Local Alignment Search Tool (BLAST) analysis against the RNAseq atlas of alfalfa^18^. For each gene, alfalfa gene sequence with highest level of homology was identified and compared with *M. truncatula* genomic sequence to identify intron-exon boundaries. We designed at least one PCR primer out of each pair, from two exons flanking an intron (Table 4).

### Quantitative Reverse Transcription-PCR (qRT-PCR) analyses

Tissue samples from roots and leaves were taken from the long term salt treatment experiment for RNA isolation. Young leaves or roots were harvested from 12 genotypes from the salt treatments and the controls with for three biological replications. Samples were frozen immediately in liquid Nitrogen and RNA was extracted using TRIzol^®^ reagent (Invitrogen, Carlsbad, CA, USA). To remove contaminating DNA, RNA was treated with DNase I following manufacturer’s instructions (Thermo Scientific, Waltham, MA, USA). The qRT-PCR experiments were carried out in a BioRad CFX96 System using iTaq^TM^ Universal SYBR^®^ Green One-Step Kit (Bio-Rad Laboratories, Hercules, CA, USA). qRT-PCR reactions were performed in total volume of 10 μl containing 100 ng total RNA, 0.75 μM of each of the primers, 0.125 μl iScript™ Reverse Transcriptase and 5 μl of 2x one-step SYBR^®^ Green Reaction mix. The PCR conditions were as follows: 50 °C for 10 min, 95 °C for 1 min, then 40 cycles of 95 °C denaturation for 10 s, 57 °C annealing for 30 s, and 68 °C extension for 30 s. Four samples were used as inter-plate controls to enable normalized expression in different plates. The alfalfa *Actin* (Act) and *glyceraldehyde 3-phosphate dehydrogenase (G3PD*) genes were used as reference in the expression analysis. Differentially expressed genes were identified by comparing the cycle threshold values of each gene to the reference gene and the relative expression differences were calculated. Melt curve analysis was used to test the amplification specificity by ramping the temperature to 95 °C for 10 s and back to 65 °C for 5 s followed by incremental increases of 0.5 °C/cycle up to 95 °C.

## Additional Information

**How to cite this article:** Sandhu, D. *et al*. Variable salinity responses of 12 alfalfa genotypes and comparative expression analyses of salt-response genes. *Sci. Rep.* **7**, 42958; doi: 10.1038/srep42958 (2017).

**Publisher's note:** Springer Nature remains neutral with regard to jurisdictional claims in published maps and institutional affiliations.

## Supplementary Material

Supplementary Information

## Figures and Tables

**Table 1:** Biomass per plant and Sodium, Potassium and Chloride contents of 12 selected mother plants for detailed salinity study.

Genotype Name	Genotype #	Biomass per plant g (rank*)	Ion composition mmol kg^−1^ dw (rank)				Classification			
			Na	Cl	K	Total-S	Biomass	Na	Cl	K
	*Cl 18.2* *dSm*^−*1*^ (*rank 1*–*60*)									
SISA 15	G01	61.7 (1)	186 (56)	203 (56)	658 (40)	132	H	L	L	M
Cuf 101-1	G02	51.5 (4)	539 (15)	393 (13)	533 (53)	135	H	H	H	L
SISA 14-1	G03	43.5 (10)	186 (55)	176 (59)	685 (34)	123	H	L	L	M
Cuf 101-2	G04	11.2 (39)	842 (1)	330 (23)	657 (41)	124	L	H	M	M
	*SO*_*4*_ *18.4* *dS m*^−*1*^ (*rank 1*–*60*)									
SISA 14-3	G08	81.4 (1)	360 (40)	197 (44)	636 (39)	197	H	L	L	M
SISA 10	G09	33.0 (25)	173 (60)	159 (56)	812 (9)	146	M	L	L	H
	*Cl 24.3* *dS m*^−*1*^ (*rank 1*–*19*)									
SW9720	G05	42.0 (1)	318 (7)	316 (10)	667 (13)	206	H	M	M	M
SISA 9	G06	32.7 (6)	106 (19)	230 (17)	513 (19)	123	H	L	L	L
SISA 14-2	G07	12.8 (11)	479 (2)	486 (2)	695 (9)	182	L	H	H	M
	*SO*_*4*_*24.7* *dS m*^−1^ (*rank 1*–*21*)									
AZ-90 ST	G10	51.7 (1)	331 (14)	161 (16)	692 (13)	161	H	L	L	M
SW9215	G11	35.3 (2)	323 (15)	382 (5)	959 (2)	182	H	L	H	H
Salado	G12	26.4 (8)	894 (4)	275 (11)	708 (11)	275	M	H	M	M

^*^Within treatments, rank 1 to higher ranks mean high to low biomass, and high to low K, Na and Cl contents.

**Table 2:** Results of a two-way analysis of variance of alfalfa clones by treatment (T), and genotype (G).

Dependent variables		T	G	T × G	Cov
Biomass		<0.001	<0.001	<0.001	<0.001
Height		<0.001	<0.001	0.047	0.015
Number of shoots		<0.001	<0.001	0.002	<0.001
Ions	Na	<0.001	<0.001	<0.001	0.185
	Cl	<0.001	<0.001	<0.001	0.301
	K	<0.001	<0.001	<0.001	0.360
	K/Na	<0.001	<0.001	<0.001	0.722
	P	<0.001	<0.001	0.010	0.636
	S	0.002	<0.001	0.001	0.490
	Ca	<0.001	<0.001	0.001	0.642
	Mg	<0.001	<0.001	0.004	0.766

Numbers represent P values. Values less than 0.05 are considered significant.

**Table 3:** Composition of the irrigation water.

Treatment	EC_iw_ (dS/m)	pH	Ion concentration (mmol_c_ L^−1^)							
			Cl^−^	SO_4_^2^	NO_3_^−^	PO_4_^3−^	Na^+^	K^+^	Ca^2+^	Mg^2+^
Control	1.97	7.5	4.27	4.62	5.03	0.04	5.41	4.7	3.01	2.85
Saline	16.6	7.3	118.1	51	5.12	0.01	134	4.7	22	20.7

**Table 4:** The list of primers used for the expression analysis.

Gene	Primer (5′–3′)
MsActin-4F	GGAATGGTCAAGGCTGGATTT
MsActin-4R	TGATTGAGCTTCATCACCAACATA
MsAKT1-1F	CGAACTAGAAAATTTAGGGATACCAT
MsAKT1-1R	CCTCCGAGTCTGTTCTAAACTTC
MsAP2-2F	AGAGGACCAAGGTCCAGAA
MsAP2-2R	TGTGTCAAATCCACCAAGATACA
MsATPase-2F	TTTGTTAATCAGGTAAAGCGATGTG
MsATPase-2R	TCCAAGTCAATGTCCGGTTG
MsAVP-1F	AGAAGTACATTGAGGCTGGTG
MsAVP-2R	GTCCAGAGGTGTCCTTCAATG
MsCDPK7-2F	CTTTACGGGTAATTGCTGAAAGTC
MsCDPK7-2R	TCCTTAAGGGTAGACCCATATCT
MsERF1-1F	GATGCTTCTCTACGGCATGAT
MsERF1-1R	CTTCTCCTTCCTCGGTTCATTT
MsG3PD-3F	CATCACAGCCACTCAGAAGAC
MsG3PD-3R	TGGAAGCACTTTGCCTACAG
MsHKT1-1F	TTCATTGTCATGATGTATCTTCCAC
MsHKT1-1R	CTGAGAGAATATAATACGATCCACTAGG
MsHSP71.1-1F	CTGAACCTACGACCTATCAACC
MsHSP71.1-1R	GATCGGTGATGCTGCTAAGA
MsHSP80.2-2F	GCTTGATTCTCAGCCAGAGT
MsHSP80.2-2R	AGGTTGTTCACCAAATCAGCTT
MsHSP90-2F	CTTCTCGCAGATCAAGGTCAA
MsHSP90-2R	GAGGAACAGCACCGAGTTT
MsHSPC025-1F	CTCCTGCTCGAGTATCTGAATAAT
MsHSPC025-1R	AATTGCTCTCTTGAAACAGTTCG
MsNHX1-2F	CAATGCCGGGTTTCAAGTAAAG
MsNHX1-2R	AGTAGCACCCGTGGTTATAATG
MsNHX2-1F	GCGCTAAACAATCCAACAAAGA
MsNHX2-1R	CCTCTCCCACATTGACTTAACC
MsOTS2-1F	AACTCAAGTCATAGCCGTTCC
MsOTS2-1R	TCTTTCAGGTGCCTCTCCTA
MsP5CS-1R	GGCTGCAGGATAATCAGTCTTT
MsP5CS-2F	GTTCTTGGTCATGCTGATGGA
MsSGF29-1F	GACAATGAAGCAAATCTTTCCACTA
MsSGF29-1R	TCTTCTTCGTGGTTGTCCTTG
MsSAL1-2F	AGTAGCCGAGGAGGATTCA
MsSAL1-2R	GTAGAAAAGTTATCTGATCCTTCATTAGC
MsSKIP1-1F	TCGCAAATAAACCCTAAATCGC
MsSKIP1-1R	AGAGCCGCCATGGTTTAG
MsSOS1-2F	TTGCTTACTACACTGCTCAAGA
MsSOS1-2R	GCGATATAAGCAATCATTTCCCAA
MsSOS2-2F	CAATATAGTCAGATTGCATGAGGTT
MsSOS2-2R	AGCTTTACCTGCTGAACAATTT
MsSOS3-2F	CTAGCTGGAATTCTTCCTTGTG
MsSOS3-2R	CAGTTACTGTGAGTGAAGTAGAG
