# Supplementary material for: Variable salinity responses of 12 alfalfa genotypes and comparative expression analyses of salt-response genes
Source: Sci Rep. 2017 Feb 22;7:42958. doi: 10.1038/srep42958 (PMC5320470; doi:10.1038/srep42958)
Supplement: Supplementary Information [file srep42958-s1.pdf]

Variable salinity responses of 12 alfalfa genotypes and comparative expression analyses of salt-response genes

Devinder Sandhu, Monica V. Cornacchione, Jorge F.S. Ferreira, Donald L. Suarez

Supplementary Fig. S1

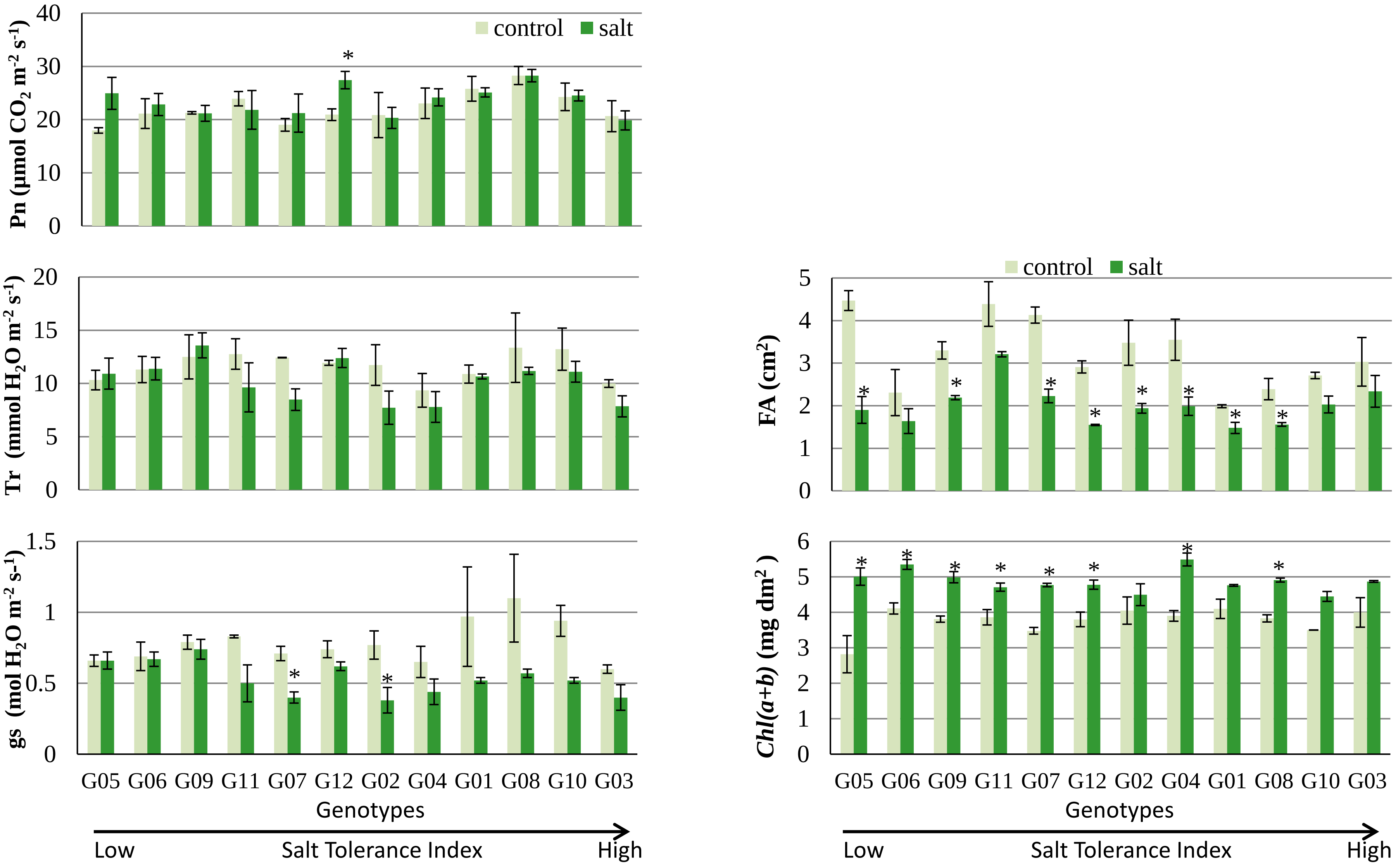

Supplementary Fig. S1. Physiological parameters of 12 alfalfa genotypes in the control and salt treatments. A, Net photosynthesis (*Pn*); B, Transpiration (*Tr*); C, stomatal conductance (*gs*); D, Foliar area (*FA*); E, Chlorophyll content (*Chl(a+b)*). Genotypes are arranged on *x*-axis in ascending order based on their ST index. Error bars represent standard error. Asterisks indicate differences (t-test $P \leq 0.05$) between the treatments for a genotype.

Supplementary Fig. S2

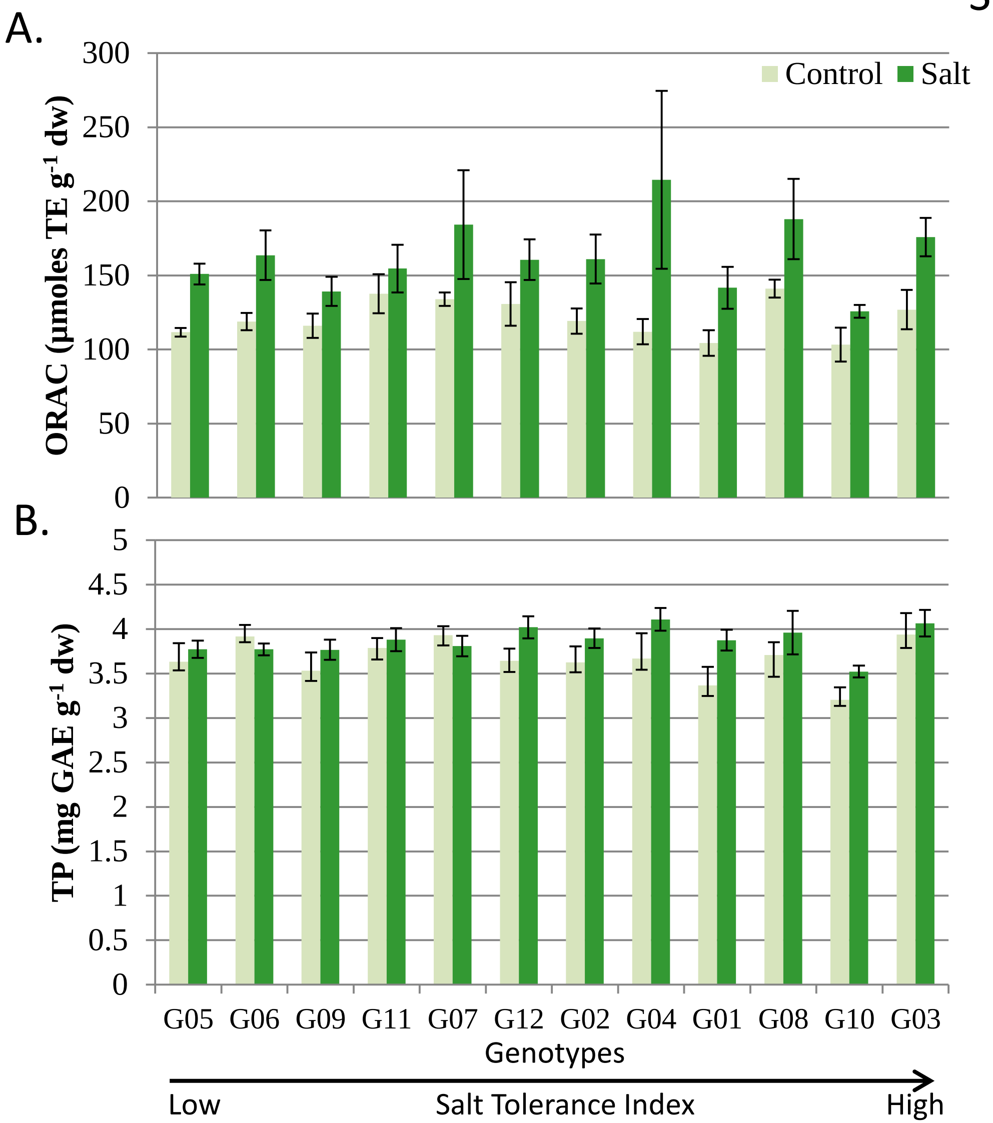

Supplementary Fig. S2. Antioxidant capacity of 12 alfalfa genotypes irrigated with control water (EC_{iw} = 1.97) and saline water (EC_{iw} = 16.6 dS m^{-1}). A, Hydrophilic oxygen radical absorbance capacity (ORAC); B, total phenolics (TP). ORAC units are micromoles of trolox equivalents per gram of dry weight (μmoles·TE·g^{-1} dw). TP units are milligrams of gallic acid equivalents per gram of dry weight (mg·GAE·g^{-1} dw). Genotypes are arranged on *x*-axis in ascending order based on their ST index. Error bars represent standard error.

Supplementary Table S1. Shoot ion concentrations of alfalfa genotypes in the control (C) and salt (S) treatments.

Genotypes	Mg mean		P mean		Fe mean		Cu mean		Mn mean		Zn mean		K/Na	
	C	S	C	S	C	S	C	S	C	S	C	S	C	S
G01	123.2	120.5	112.3	98.5	94.0	76.5	12.0	9.4	19.1	35.0	23.4	30.9	21.4	5.1
G02	128.5	118.0	101.7	99.8	81.3	72.3	9.0	9.7	20.3	27.6	23.3	34.0	9.7	1.4
G03	136.0	140.8	102.7	93.4	88.4	77.5	10.8	9.2	17.7	31.1	24.9	31.0	18.8	4.7
G04	134.0	102.6	104.9	95.3	90.6	71.8	9.5	10.4	25.5	42.8	25.6	31.5	9.5	1.5
G05	141.5	146.8	108.6	109.9	93.5	91.0	15.7	10.6	18.7	32.7	28.8	32.1	14.1	2.5
G06	173.3	180.0	104.4	97.0	83.5	69.7	12.3	10.6	21.3	37.5	25.9	31.4	22.0	8.0
G07	142.3	122.9	117.1	98.4	101.4	76.9	11.6	11.7	21.0	30.5	26.2	31.8	10.7	1.8
G08	164.9	147.3	101.5	92.4	87.0	77.6	8.8	9.7	18.6	27.3	23.6	34.0	14.1	3.2
G09	117.5	116.3	121.3	101.2	74.9	66.0	12.6	9.0	18.2	41.0	26.2	31.3	24.3	8.2
G10	150.0	126.2	95.3	87.9	84.4	75.5	9.5	9.5	18.8	27.2	21.7	26.4	14.4	2.9
G11	119.4	98.9	100.5	97.7	100.1	83.3	9.3	16.1	20.0	35.6	25.5	35.2	15.8	2.8
G12	132.3	117.2	103.4	93.9	76.8	66.6	11.4	9.1	16.4	24.0	27.4	29.3	7.1	1.5
Average	138.6	128.1	106.1	97.1	88.0	75.4	11.0	10.4	19.6	32.7	25.2	31.6	15.1	3.6
